# Characterisation of *in vitro* resistance selection against second-/last-line antibiotics in methicillin-resistant *Staphylococcus aureus* ATCC 43300 strain

**DOI:** 10.1093/jacamr/dlaf108

**Published:** 2025-06-23

**Authors:** Anggia Prasetyoputri, Miranda E Pitt, Minh Duc Cao, Soumya Ramu, Angela Kavanagh, Alysha G Elliott, Devika Ganesamoorthy, Ian R Monk, Timothy P Stinear, Matthew A Cooper, Lachlan J M Coin, Mark A T Blaskovich

**Affiliations:** Centre for Superbug Solutions, Institute for Molecular Bioscience, The University of Queensland, Brisbane, Australia; Research Centre for Applied Microbiology, National Research and Innovation Agency, Cibinong, Indonesia; Centre for Superbug Solutions, Institute for Molecular Bioscience, The University of Queensland, Brisbane, Australia; Australian Institute for Microbiology and Infection, University of Technology Sydney, Ultimo, New South Wales, Australia; Centre for Superbug Solutions, Institute for Molecular Bioscience, The University of Queensland, Brisbane, Australia; Centre for Superbug Solutions, Institute for Molecular Bioscience, The University of Queensland, Brisbane, Australia; Centre for Superbug Solutions, Institute for Molecular Bioscience, The University of Queensland, Brisbane, Australia; Centre for Superbug Solutions, Institute for Molecular Bioscience, The University of Queensland, Brisbane, Australia; Centre for Superbug Solutions, Institute for Molecular Bioscience, The University of Queensland, Brisbane, Australia; Department of Microbiology and Immunology, The University of Melbourne at The Peter Doherty Institute for Infection and Immunity, Melbourne, Victoria, Australia; Department of Microbiology and Immunology, The University of Melbourne at The Peter Doherty Institute for Infection and Immunity, Melbourne, Victoria, Australia; Centre for Superbug Solutions, Institute for Molecular Bioscience, The University of Queensland, Brisbane, Australia; Sitala Bio Ltd., Unit D6, Grain House, Mill Ct., Shelford, Cambridgeshire CB22 1LD, UK; Centre for Superbug Solutions, Institute for Molecular Bioscience, The University of Queensland, Brisbane, Australia; Department of Microbiology and Immunology, The University of Melbourne at The Peter Doherty Institute for Infection and Immunity, Melbourne, Victoria, Australia; Centre for Superbug Solutions, Institute for Molecular Bioscience, The University of Queensland, Brisbane, Australia

## Abstract

**Background and objectives:**

The increasing occurrence of MRSA clinical isolates harbouring reduced susceptibility to mainstay antibiotics has escalated the use of second and last line antibiotics. Hence, it is critical to evaluate the likelihood of MRSA developing clinical resistance to these antibiotics. Our study sought to characterize the development of resistance to vancomycin (VAN), daptomycin (DAP) and linezolid (LZD) in MRSA ATCC 43300 *in vitro* and further determine the mechanisms underpinning resistance.

**Methods:**

MRSA was exposed to increasing concentrations of VAN, DAP and LZD for 20 days, with eight replicates for each antibiotic conducted in parallel. The resulting day 20 (D20) isolates were subjected to antimicrobial susceptibility testing, whole genome sequencing, autolysis assays, and growth curves to determine bacterial fitness.

**Results:**

Exposure to VAN or LZD for 20 days resulted in a subtle 2-fold increase in the MIC, whereas DAP exposure yielded DAP-non-susceptible isolates with up to 16-fold MIC increase. The MIC increase was accompanied by variable changes in relative fitness and reduced resistance to autolysis in some isolates. D20 isolates harboured mutations in genes commonly associated with resistance to the respective antibiotics (e.g. *walK* for VAN, *mprF* and *rpoB* for DAP, *rplC* for LZD), along with several previously unreported variants. Introduction of key mutations to these identified genes in the parental strain via allelic exchange confirmed their role in the development of resistance.

**Conclusions:**

*In vitro* selection against VAN, DAP or LZD resulted in the acquisition of mutations similar to those correlated with clinical resistance, including the associated phenotypic alterations.

## Introduction

MRSA has retained its ranking as a ‘High’ priority pathogen on the revised 2024 WHO Bacterial Priority Pathogens List^1^ and has been identified as one of the leading causes of global infections^2,3^ and economic burden.^4^ The glycopeptide antibiotic vancomycin (VAN) has been a mainstay treatment for MRSA-related infections.^5,6^ Although complete resistance against VAN in MRSA is currently exceptional,^7^ its efficacy is compromised by the increasing incidence of vancomycin-intermediate *S. aureus* (VISA) and heterogeneous VISA (hVISA).^8,9^ Treatment failures and poor clinical outcomes in glycopeptide-treated patients have been associated with reduced VAN susceptibility^7,10,11^ and VAN tolerance.^12^ Furthermore, emerging resistance to alternative MRSA antibiotics, such as linezolid (LZD)^13,14^ and daptomycin (DAP)^15^ also adds to the current burden, and highlights the importance for discovery of new MRSA antibiotics.

Clinical resistance in MRSA to VAN, DAP and LZD and their underlying genetic basis has been extensively investigated^7,16–18^ Elucidation of the genomic basis of resistance has been conducted in clinical isolates^19,20^ as well as laboratory-derived strains.^21,22^ Collectively, these have revealed pathways where mutations can occur upon exposure to antibiotics and could potentially assist in designing new antibiotics that could bypass existing resistance mechanisms.

New MRSA antibiotics ideally would have a low propensity to select for clinical resistance. To enable development of such antibiotics, a comprehensive understanding of the nature of resistance mechanisms that might impair their efficacy, and the rate at which that resistance arises, is essential. Availability of *in vitro* resistance selection that could reveal genomic and phenotypic changes similar to those found in clinical settings would be a valuable tool for predicting mutations and monitoring the progression of resistance before a candidate antibiotic progressed to the clinic. As an example, laboratory-derived MRSA with reduced susceptibility to DAP were generated via resistance selection^23–25^ and certain genomic variants potentially associated with resistance development were identified. Resistance selection could also provide clues to potential cross-resistance between antibiotics having the same cellular target^26^ or give insights into antibiotic resistance mechanisms.^27^

This study aimed to investigate the usefulness of using *in vitro* selection of resistance against VAN, DAP or LZD in MRSA to predict mutations similarly identified in clinical isolates. We selected an MRSA strain (ATCC 43300) which is susceptible to VAN, DAP and LZD. ATCC 43300 was serially passaged in the presence of increasing concentrations of each antibiotic and the development of resistance was monitored over 20 days. Genomic and phenotypic alterations were elucidated along with propensities for cross-resistance between each antibiotic studied. These investigations are expected to provide further insight into resistance development in MRSA and tools for predicting potential clinical resistance in the context of novel antibiotic discovery.

## Materials and methods

### Bacterial strains and growth conditions

MRSA ATCC 43300 isolates were sourced from the American Type Culture Collection (ATCC; Manassas, USA). ATCC 43300 (housed at the University of Queensland—MIC: VAN = 1 mg/L; DAP = 0.5 mg/L; LZD = 2 mg/L) was used for *in vitro* resistance selection, subsequent phenotypic assays and genome sequencing. Another ATCC 43300 (University of Melbourne—MIC: VAN = 1 mg/L; DAP = 0.125 mg/L) was used in allelic exchange assays. Bacterial isolates were stored in 20% v/v glycerol at −80°C. ATCC 43300 was grown in cation-adjusted Muller Hinton Broth (Ca-MHB, Oxoid) at 37°C, 220 rpm, unless otherwise indicated.

### Resistance selection

*In vitro* resistance selection was conducted^28^ for VAN, DAP or LZD, with a detailed procedure in Supplementary data (available as Supplementary data at *JAC-AMR* Online). Corning^®^ non-binding surface 96-well plates (Sigma-Aldrich) were used to minimize binding of antibiotics to test plate.^29^ (Supplementary material). Following 20 days of antibiotic passaging, isolates were further grown for 5 days (Day 25 (D25)) without antibiotic to assess stability of resistance.

### Antimicrobial susceptibility testing

The MIC was determined by the broth microdilution method according to CLSI guidelines^30^ using Ca-MHB (Supplementary data). Stock solutions of VAN, DAP and LZD were each prepared and dissolved in water at 1280 mg/L concentration, giving a range of concentrations tested between 64 and 0.03 mg/L. Susceptibility of all D20 isolates was assessed against their respective selecting antibiotic after the resistance selection experiment was completed, as well as against all antibiotics (VAN, DAP and LZD) to assess potential cross-resistance. MIC breakpoints were determined using standards from EUCAST version 15.0,^31^ where resistance was defined as MIC ˃ 2 mg/L for VAN, MIC ˃ 1 mg/L for DAP, and MIC ˃ 4 mg/L for LZD.

### DNA extractions and library preparation

Aliquots (10 µL) of glycerol stocks of initial day (day 0/D0) and day 5, 10, 15 and 20 isolates were grown overnight in Ca-MHB supplemented with antibiotics (up to half MIC recorded) (Table S1). Cells from 4 mL of culture were centrifuged (14 000 rpm, 2 min) and DNA extracted using DNeasy Blood and Tissue Kit (QIAGEN) according to manufacturer’s instructions. Following quantification with Qubit^®^3.0 (Thermo Fisher Scientific), a library preparation with 1 ng DNA input was conducted using Nextera XT Kit (Illumina). Library preparation of the D0 isolate was performed with the SQK-LSK109 kit (Oxford Nanopore Technologies, ONT) and sequenced on an R9 flow cell. DNA fragmentation was determined with a TapeStation 4200 (Agilent).

### Sequencing and analysis

All DNA libraries were run on Illumina HiSeq2500 with 150 bp paired-end reads and ≥ 100× coverage. The reference D0 isolate was sequenced on a MinION (ONT), base-called using Guppy 2.3.7 and a complete hybrid assembly (Illumina, ONT reads) generated via Unicycler v0.3.7.^32^ Illumina reads were trimmed using Trimmomatic v0.27,^33^ assembled using SPAdes v3.10.1^34^ and annotated using Prokka v1.12.^35^ Trimmed reads were mapped to D0 using BWA-MEM^36^ with default settings. GATK Unified Genotyper^37^ was used to call single-nucleotide polymorphisms (SNPs) and small insertion and deletions (indels) from high-quality reads and impact of non-synonymous variants was annotated using snpEff v4.1,^38^ followed by further quality filtering with SnpSift.^39^ Snippy v4.6.0 was also implemented to confirm Day 20 variants.^40^

### Autolysis

Selected D20 isolates with increased VAN MIC were subjected to Triton X-induced autolysis.^41^ Briefly, overnight cultures were prepared in Brain Heart Infusion (BHI) broth (Oxoid) by inoculating 10 µL of glycerol stock into 4 mL BHI at 37°C, 200 rpm for 16–20 h. Subcultures were prepared in 50 mL BHI (1:40) and mid log-phase cultures (OD_600_ = 0.4–0.6) were pelleted (13 000 rpm, 15 mins, 4°C). Cells were washed twice with ice-cold sterile water before resuspension in freshly prepared 0.05 M Tris-HCl (pH 7.2) containing 0.05% (v/v) Triton X-100 (Sigma Chemical Co., St. Louis, Mo.) and incubated at 37°C, 200 rpm. Absorbance was monitored every 30 min for 5 h. Results are presented as percentage of OD_600_ decrease after 5 h relative to the initial OD_600_. Controls include D0, MRSA VISA (NRS1; Mu50) ATCC 700699 and MSSA ATCC 29213.

### Bacterial fitness

Relative bacterial fitness was determined by generating growth curves to obtain the average doubling time (DT) following a previous method,^42^ with some modifications (Supplementary data).

### Allelic exchange

To validate if mutations confer resistance, selected genes from VAN- and DAP D20 isolates were incorporated into WT MRSA ATCC 43300 via allelic exchange as previously described^43^ (Supplementary data, Table S2). All WT and resulting mutant strains underwent antibiotic susceptibility testing as described above.

### Data availability

Nucleotide sequences including genome assemblies (.fasta) and base-called (.fastq) data for both Illumina and ONT are deposited under NCBI BioProject: PRJNA986267 (www.ncbi.nlm.nih.gov/bioproject/986267).

## Results

### Acquisition and retention of *in vitro* resistance differed between antibiotic treatments

**Figure 1.**
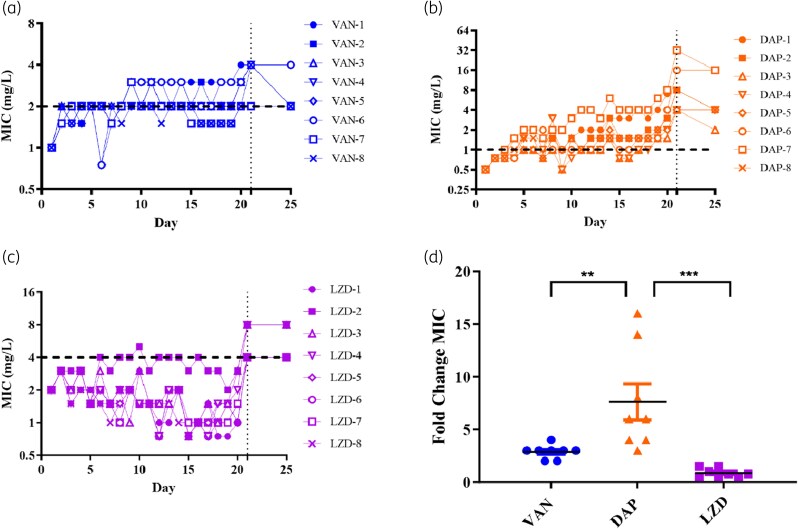
ATCC 43300 acquisition and stability of resistance towards vancomycin, DAP, and LZD. Average MIC values were plotted for (a) VAN (b) DAP and (c) LZD treated isolates. Horizontal dotted line represents clinical breakpoints for respective antibiotics based on EUCAST v15.0. Vertical line at D21 includes re-cultured D20 glycerol stocks and subsequent passaging for 5 days with no antibiotics. D25 value indicates highest MIC detected for replicates (*n* = 4). (d) Fold change in MIC values of day 20 isolates compared with day 0 (mean ± SD; *n* = 8). Statistical significance was determined by one-way ANOVA with Tukey’s multiple comparisons test (*P* < 0.05 was considered as significant; ** *P* = 0.0080; *** *P* = 0.0003). (a)–(c) *y*-axis graphed as log (2).

The susceptibility profiles of D20 isolates varied greatly between VAN, DAP and LZD following resistance selection (Figure 1a–c). Initially, few VAN isolates surpassed the clinical breakpoint after 20 days of VAN exposure; however, upon re-culturing of vancomycin D20 glycerol stocks, all but one (VAN-7) had MIC of 4 mg/L (Figure 1a). On the other hand, all of the DAP isolates reached the clinical breakpoint of 1 mg/L, with the re-cultured D20 glycerol stocks yielding MICs of 2–4 mg/L (Figure 1b). While VAN and LZD isolates experienced <4-fold increases in MIC after 20 days, DAP isolates acquired up to 16-fold increases (Figure 1d). Five days of passaging without antibiotics to discern resistance stability were conducted (Figure 1a–c). Over half of the VAN D20 resistant isolates regained susceptibility, and all DAP isolates retained resistance. Whilst all LZD D20 isolates were identified as susceptible, re-culturing glycerol stocks revealed three (LZD-2, LZD-3, LZD-4) exhibited resistance (8 mg/L) which was stable for the five antibiotic-free passages. Several variants owing to heterogeneity were detected within D0 and across numerous D20 isolates including *recX* (T737G), *agrA* (179_185dupTTCAACT) and *agrC* (518_519insATCACTCGCATCAATTTGCATATTCGCAAATTGATGC).

### Genomic alterations reflect pathways associated with the antibiotics’ target

VAN isolates exhibited chromosomal mutations in genes associated with cell wall synthesis and metabolism, such as *walK*, *atl_3* and *korB*, as well as those involved in protein synthesis (*pheS*) and virulence regulation (*tlyC* and *rny*) (Table 1). A deletion in bifunctional autolysin-encoding *atl_3* gene was detected in VAN-5 and VAN-6. No non-synonymous variants were detected in VAN-8; however, resistance was only briefly present in the D20 re-culture and quickly reverted to susceptible (Figure 1a). The frequency these mutations appeared over the time course was monitored (Table S3). Some of the mutations appeared as early as day 5 and established well before D20. Interestingly, the mutations affecting the protein synthesis pathway (*rny* and *pheS*) appeared at a later time and subpopulations having no mutations still existed by D20.

**Table 1.** Non-synonymous variants detected in D20 isolates and the resulting phenotype

Strain^a^	Gene name^b^	Protein name	Nucleotide change	Protein change	Phenotype^c^ (MIC, *fold increase from D0*)
VAN-1	*tlyC*	Hemolysin C	C391T	Pro131Ser	R (4, *2*)
	*Rny*	Ribonuclease Y	G896A	Arg299Lys	
VAN-2	*korB*	2-oxoglutarate oxidoreductase subunit KorB	A386G	Gln129Arg	R (4, *2*)
VAN-3	***walK***	Sensor protein kinase WalK	C1095A	Asp365Glu	R (4, *2*)
VAN-4	*pheS*	Phenylalanine—tRNA ligase alpha subunit	C17T	Thr6Ile	R (4, *2*)
VAN-5	*atl_3*	Bifunctional autolysin	579delA	Glu193fs	R (4, *2*)
VAN-6	*atl_3*	Bifunctional autolysin	579delA	Glu193fs	R (4, *2*)
DAP-2	*ptsI*	Phosphoenolpyruvate-protein phosphotransferase	C1399T	Arg467Cys	R (8, *8*)
DAP-3	*epsJ*	Putative glycosyltransferase EpsJ	G527T	Ser176Ile	R (4, *4*)
	*ltaS*	Lipoteichoic acid synthase	C296T	Thr99Met	
DAP-4	*epsJ*	Putative glycosyltransferase EpsJ	G527T	Ser176Ile	R (4, *4*)
	*ltaS*	Lipoteichoic acid synthase	C296T	Thr99Met	
	*ychF*	Ribosome-binding ATPase YchF	426delA	Lys142fs	
DAP-5	*lacC_2*	Tagatose-6-phosphate kinase	442_450delGCACAAATT	Ala148_Ile150del	R (4, *4*)
DAP-6	*rsmG*	Ribosomal RNA small subunit methyltransferase G	G238T	Ala80Ser	R (16, *16*)
DAP-7	*rsmG*	Ribosomal RNA small subunit methyltransferase G	G238T	Ala80Ser	R (32, *32*)
	***mprF***	Phosphatidylglycerol lysyltransferase	C1010T	**Ser337Leu**	
	*lacC_2*	Tagatose-6-phosphate kinase	365dupA	Asn122fs	
DAP-8	***rpoB***	DNA-directed RNA polymerase subunit beta	C2146T	Arg716Cys	R (8, *8*)
	*lacC_2*	Tagatose-6-phosphate kinase	C833T	Ala278Val	
	*proS*	Proline–tRNA ligase	T662G	Ile221Ser	
LZD-2	***rplC***	50S ribosomal protein L3	**G463C**	**Gly155Arg**	R (8, *2*)
LZD-3	***rplC***	50S ribosomal protein L3	**G463C**	**Gly155Arg**	R (8, *2*)
LZD-4	***rplC***	50S ribosomal protein L3	**G463C**	**Gly155Arg**	R (8, *2*)

Affected gene(s) and/or mutation(s) previously reported in the literature from clinical and/or laboratory-derived isolates are marked in bold.

^a^VAN, Vancomycin; DAP, Daptomycin; LZD, Linezolid; -, replicate number.

^b^High and moderate impact variants having read depth ≥ 50 as determined by snpEff. Illumina sequencing produced > 100-fold coverage. fs = frameshift mutation; del = deletion, del = deletion.

^c^Resistant (R) based on median MIC (mg/L) and breakpoint according to EUCAST. Fold-increase MIC from day 0 is italicized.

DAP D20 isolates exhibited more mutations compared with VAN, and a greater diversity of pathways were affected (Table 1). Genomic variants were found in genes having a role in cell wall synthesis (*ltaS* and *epsJ*), membrane phospholipid production (*mprF*), protein synthesis pathway (*ychF*, *rpoB*, *proS* and *rsmG*), as well as glucose (*lacC_2*) and carbohydrate (*ptsI*) metabolism pathways. Similar to VAN, *recX* (M246R) was also detected in DAP-3 and DAP-4. All isolates became resistant to DAP (4–32 mg/L). Development of DAP-non-susceptibility (DAP-NS) was likely brought about by an accumulation of mutations, half of which appeared after day 10 (Table S3). The appearance of certain mutations did correspond with increases in MIC. For example, the *mprF* mutation was initially detected at day 10 in the DAP-7 and the MIC increased significantly from day 10 onward (Figure 1b, Table S3). Similarly, the *rpoB* mutation in the DAP-8 started to appear from D15 and by D20 its MIC increased by 2-fold (Figure 1b, Table S3).

Variants detected in LZD isolates were minimal and only impacted *rplC* (G155R), which affects the 50S subunit ribosomal protein L3. Only three isolates harboured this mutation and had a resistant phenotype (MIC: 8 mg/L) after re-culturing D20. This mutation appeared between D10 and 15 in these isolates (Table S3).

### Potential cross-resistance between VAN and DAP isolates

**Figure 2.**
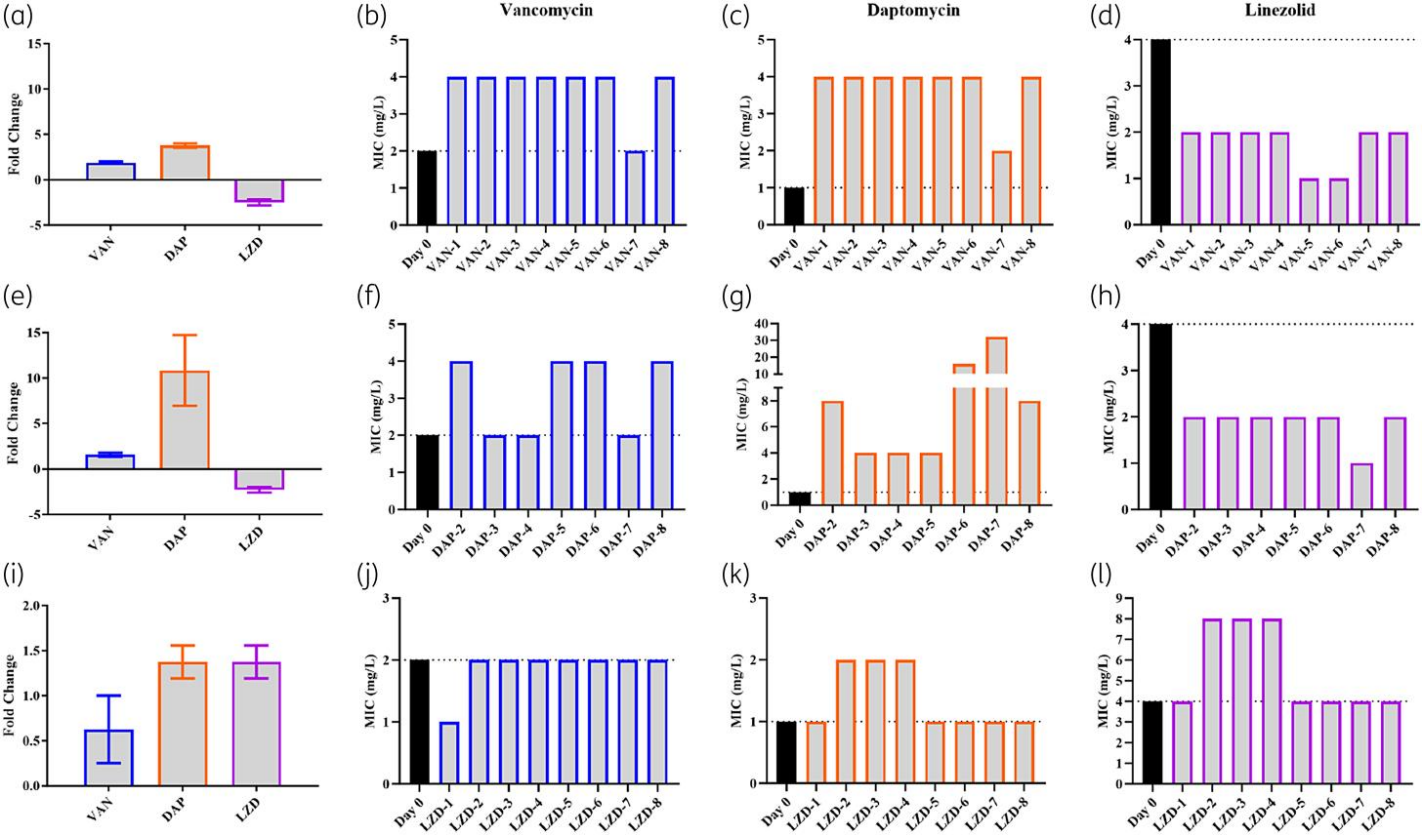
Cross-resistance between D20 isolates. (a) VAN D20 isolate fold change for all three antibiotics. MICs of VAN D20 isolates against (b) VAN, (c) DAP, and (d) LZD. (e) DAP D20 isolate fold change. MICs of DAP D20 isolates against (f) VAN, (g) DAP, and (h) LZD. (i) LZD D20 isolate fold change for all three antibiotics. MICs of LZD D20 isolates against (j) VAN, (k) DAP, and (l) LZD. Fold change represented as average (*n* = 8)±SEM and MICs performed for *n* ≥ 4. Dotted line indicates breakpoints (EUCAST) and all experiments used re-cultured −80°C D20 glycerol stocks.

Nearly all VAN D20 isolates exhibited reduced susceptibility to DAP, with the exception of VAN-7 (Figure 2a and b) (Table S4). All VAN isolates had up to 4-fold increase in DAP MICs that resulted in DAP-NS phenotypes (Figure 2c), suggesting cross-resistance between VAN and DAP. Conversely, these D20 isolates exhibited a 2-fold MIC decrease against LZD, with all isolates remaining well below the breakpoint (Figure 2d).

DAP D20 isolates exhibited >10-fold increases in MIC against DAP (Figure 2e and g). Half of the isolates also had elevated VAN MICs over its breakpoint, while the other half exhibited <2-fold MIC changes (Figure 2e and f). However, the DAP D20 isolates remained susceptible to LZD (Figure 2e and h).

LZD D20 isolates demonstrated a slight elevation in MIC against the three antibiotics tested, only by <2-fold overall (Figure 2i). All D20 isolates remained susceptible to VAN (Figure 2j), while three out of eight isolates (LZD-2, LZD-3 and LZD-4) exhibited a DAP-NS phenotype, reaching a DAP MIC of 2 mg/L (Figure 2k). Although this result suggested potential cross-resistance between LZD and DAP, the LZD MIC increase was only within a 2-fold dilution. The same three isolates also had elevated MICs against LZD (Figure 2l).

### Variable bacterial fitness costs evident for differing antibiotic exposure

**Figure 3.**
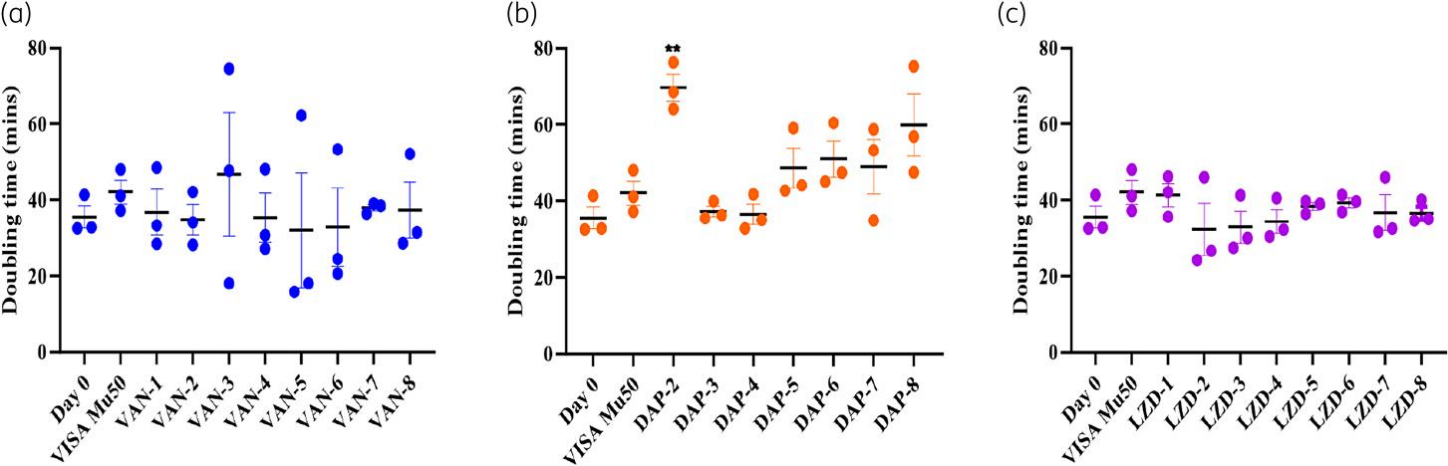
Relative bacterial fitness of D20 isolates. DT (in minutes) of VAN (a), DAP (b) and LZD (c) D20 isolates were calculated to measure the relative fitness compared with the initial D0 isolate. Fitness determined using three biological replicates (mean ± SD). Significant difference as measured by a two-tailed Welch’s *t*-test (*P* < 0.05) between D20 versus D0 is shown with asterisks (**P = 0.0020).

Average DT for each D20 isolate was calculated to assess whether mutations had any impact on the relative bacterial fitness. VAN D20 isolates had a large variability of DT within the biological replicates (Figure 3a) with no significant difference. DAP D20 isolates were found to exhibit DTs longer than that of D0 isolate, though only DAP-2 was significantly different (DT = 69.6 ± 6.1 mins, a 96% increase) (Figure 3b, Table S5). Meanwhile, no significant variation in DTs was found in LZD isolates (Figure 3c, Table S5).

### Differing degrees of autolysis were observed for VAN and DAP D20 isolates

**Figure 4.**
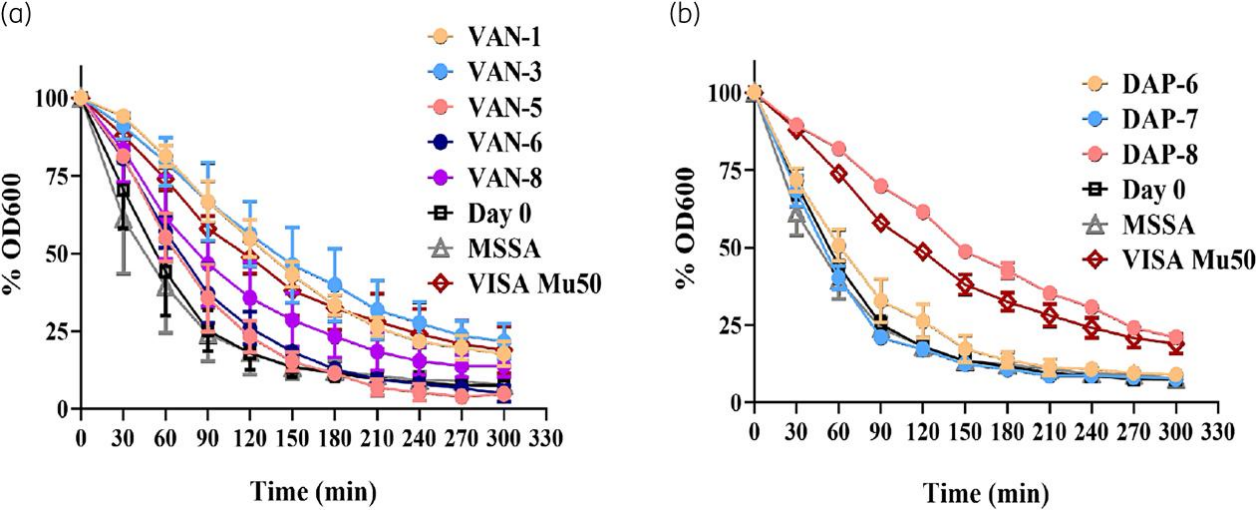
Triton-X-induced autolysis assay on selected VAN and DAP D20 isolates. Individual plotted percentage of optical density decrease for D20 isolates exhibiting reduced susceptibility. Isolates selected exhibited an elevated MIC as well as mutations in pathways associated with changes in autolysis. (a) VAN isolates VAN-1, VAN-3, VAN-5, VAN-6 and VAN-8 (MIC 4 mg/L); (b) DAP isolates DAP-6, DAP-7 and DAP-8 (MIC 16, 32, and 8 mg/L, respectively). Autolysis measured over 5 h. Presented as the percentage decrease in optical density relative to initial D0 (mean ± SD). Controls include VISA Mu50 (known tolerance to autolysis) and MSSA (sensitive to autolysis). Data was obtained from three independent experiments.

D20 isolates were selected based on their increased MIC to VAN (MIC 4 mg/L) or to DAP (MIC 8–32 mg/L), as well as the presence of mutation/s that are usually associated with reduced autolysis. The rate of Triton-X-induced autolysis was compared with D0, MSSA and a VISA Mu50 isolate known to exhibit reduced autolysis. VAN-1 and VAN-3 exhibited a slower rate of OD_600_ decrease compared with D0 with rates similar to the VISA Mu50 (Figure 4a). VAN-8 also had a slower rate of autolysis compared with D0 (50% OD_600_ decrease after 90 min) but not as slow as the VISA Mu50 (50% OD_600_ decrease after 150 min). In contrast, VAN-5 and VAN-6 had a slightly delayed rate of autolysis compared with D0, though not as slow as the VISA strain, having a 50% OD_600_ decrease between 60 and 90 min. DAP-6 and DAP-7 exhibited similar trends in their resistance to autolysis, having similar rates of OD_600_ decrease to D0 (Figure 4b). Conversely, DAP-8 showed a higher resistance to autolysis compared with D0, exhibiting a similar, if not higher, rate of OD_600_ decrease to the VISA Mu50. No LZD isolates were tested for reduced autolysis due to the lack of mutations associated with autolytic activity.

### Certain variants contribute to the development of resistance

To ascertain whether variants contribute to resistance, we generated mutants of genes harbouring both known and novel mutations via allelic exchange. Four mutants were successfully generated: *walK* (Asp365Glu), *mprF* (Ser337Leu), *rsmG* (Ala80Ser) and *ptsI* (Arg476Cys) (Table 2). Although *rsmG* and *ptsI* have not been reported to be associated with drug resistance in MRSA, *rsmG* (Ala80Ser) was the only change detected in DAP-6 (MIC: 16 mg/L) and present in DAP-7 (MIC: 32 mg/L) (Table 1). The *ptsI* (Arg476Cys) variant was found in DAP-2 exhibiting severely reduced growth rate (Figure 3). The generation of *rpoB* (Gly716Cys) and *rplC* (Gly155Arg) mutants was unsuccessful. As the LZD mutation in *rplC* was known to cause resistance, this was excluded.

**Table 2.** MIC of mutants generated by allelic exchange

Strain^a^	Vancomycin MIC^b^ (mg/L)	Fold-change MIC^c^	Daptomycin MIC^b^ (mg/L)	Fold-change MIC^c^	Parental D20 isolate mutation was detected
MRSA ATCC 43300	1^S^	—	0.125^S^	—	—
*mprF* (Ser337Leu)	2^S^	2	2^R^	16	DAP-7
*rsmG* (Ala80Ser)	1^S^	—	0.25^S^	2	DAP-6, DAP-7
*ptsI* (Arg476Cys)	1^S^	—	0.25^S^	2	DAP-2
*walK* (Asp365Glu)	2^S^	2	0.25^S^	2	VAN-3

^a^ATCC 43300 isolate (University of Melbourne) was transformed via allelic exchange (MIC: VAN—1 mg/L, DAP—0.125 mg/L) and mutations are depicted as the gene(s) impacted and the respective amino acid changes in brackets.

^b^Susceptibility of transformed isolates and parental ATCC 43300 to VAN and DAP was determined by MIC in Ca-MHB (*n* = 4), where R = resistant and S = susceptible (EUCAST).

^c^Fold-change MIC was calculated based on the MIC of transformed compared with parental ATCC 43300.

The *walK* (Asp365Glu) mutant had a 2-fold increase in VAN MIC, which is consistent with our observations in VAN-3 (Table 2). The 2-fold increase in DAP MIC suggested potential cross-resistance between VAN and DAP, despite it being less than observed in VAN-3 (4-fold increase in DAP MIC compared with D0) (Table 2). Similarly, the *mprF* (Ser337Leu) mutant exhibited a 16-fold increase in DAP MIC and a 2-fold increase in VAN MIC, suggesting cross-resistance and providing evidence of the role of *mprF* (Ser337Leu) in DAP-NS phenotype observed in DAP-6.

*rsmG* (Ala80Ser) and *ptsI* (Arg476Cys) had a 2-fold increase in DAP MIC compared with the parental MRSA, but had no change in their VAN MIC. This is different from the MIC determination of the D20 resistance selection isolates, where DAP-2 and DAP-6 had 8-/16-fold increase in DAP MIC compared with D0, respectively. The *rsmG* (Ala80Ser) mutation may partially contribute to DAP resistance in DAP-7, but further studies are required to unravel the 16-fold increase in DAP-6. Similarly, the precise role of the *ptsI* (Arg476Cys) in contributing to the DAP-NS phenotype in DAP-2 will also require further studies.

## Discussion

*In vitro* resistance selection assays provide key insights into the mechanisms bacteria can employ to develop resistance and can mimic genomic changes detected in a clinical setting. This was evident in our study which exposed MRSA to 20 days of VAN, DAP or LZD. Subsequent phenotypes associated with pathways impacted such as fitness and autolysis correlated with clinical isolates.

The WalKR two-component regulator, crucial for cell wall synthesis and metabolism, is strongly associated with VAN resistance.^44,45^ Our study also confirmed (VAN-3, *walK* (Asp365Glu), MIC 4 mg/L) and validated this with allelic exchange. The Asp365Glu mutant has yet to be reported and impacts the PAS domain. This variant is within one residue of a lab-derived VISA strain harbouring a *walK* mutation (His364Arg) exhibiting decreased autolytic activity and longer DTs.^46^

DAP clinical resistance can be associated with genes *mprF* and *rpoB*^47–52^ and *in vitro* assays,^23,25,53^ consistent with our findings. *mprF* plays a role in lysinylation of cell membrane phosphatidylglycerol, generating lysyl-PG and its translocation to the outer cell membrane. Mutations impacting the *mprF* gene result in a gain-in-function phenotype that includes increased cell membrane charge, leading to repulsion of the calcium-DAP complex.^54^ Interestingly, this particular Ser337Leu mutation in DAP-7 has been reported in several studies associated with the DAP-NS phenotype in clinical and laboratory-derived strains.^47,49,53,55–67^ DAP-7 was found to have the highest increase in DAP MIC (32 mg/L), but remained susceptible to VAN (MIC: 2 mg/L) as well as a longer DT, similar to previous studies.^49,68^ DAP-8 harboured the *rpoB* (Arg716Cys) variant and resulted in reduced susceptibility to both DAP (MIC: 8 mg/L) and VAN (MIC: 4 mg/L), suggesting cross-resistance. Although the exact mutation has not been reported, the concurrent reduced susceptibility to DAP and VAN has been reported in other *rpoB* perturbations.^48,69^  *rpoB* mutations can exhibit a VISA phenotype,^70^ which was evident in our study as DAP-8 had an autolysis trend similar to VISA Mu50.

Mutations in *rplC* have been detected in clinical LZD-resistant isolates^71,72^ LZD selection yielded a Gly463Cys mutation in *rplC* encoding the 50S ribosomal protein L3. This exact amino acid change has previously been found in an *in vitro* assay using MSSA.^73^ This mutation is located within the peptidyl transferase centre (PTC) of the 50S ribosomal subunit, and based on structural studies, can interfere with a conserved part of the PTC and cause reduced binding affinity.^73^ Resistance against LZD has been largely attributed to mutations in the domain V of 23S rRNA,^15,74^ but mutations in ribosomal protein L3 encoded by *rplC* (as well as ribosomal protein L4 encoded by *rplD*), are also known to be associated with resistance.^74^

DTs for all D20 isolates revealed DAP-2 exhibiting a severely impaired growth, in which an Arg476Cys mutation was identified in *ptsI*. *ptsI* is part of the phosphotransferase system (PTS), and mutations in the PTS system have been implicated in heightened DAP resistance in *Enterococcus faecium*.^75^ Mutation in *ptsI* is also associated with fosfomycin resistance in *E. coli* that confers a fitness cost.^76^ Further experimentation is needed to confirm whether this *ptsI* mutation is responsible for impaired fitness. Our allelic exchange assay revealed a 2-fold increase in DAP MIC, suggesting a role in MRSA resistance.

Cross-resistance between VAN and DAP has often been documented with genes, such as *walK* and *mprF*, which confer both VISA and DAP-NS phenotypes.^53,77–79^ For example, mutations affecting *walK* have been shown to cause cross-resistance,^80^ similar to our observations with VAN-3 with DAP MIC of 4 mg/L. This is consistent with reports on DAP-NS accompanied by reduced susceptibility to VAN in the clinic,^81–83^ and DAP-NS phenotypes emerging from VAN treatment.^83^ This study found that 50% of VAN D20 isolates became DAP-NS. Similarly, some of DAP D20 had elevated VAN MIC, as exemplified by DAP-6 harbouring *mprF* (Ser337Leu) causing a 2-fold elevation in VAN MIC. The concurrent evolution of resistance to both VAN and DAP may be due to common pathway/s,^84^ which may enable resistance to newly developed antibiotics harbouring a similar mechanism of action.^85^ All VAN D20 isolates became DAP-NS, but DAP isolates exhibited variable VAN susceptibility profiles. Hence, cross-resistance is likely dependent on the genes and their mutations. Notably, the DAP-7 S337L *mprF* mutation that remained VAN susceptible. Ruzin *et al*.^86^ demonstrated that *mprF* impairment could lead to increased VAN susceptibility.^86^ Additionally, VAN reduced susceptibility could be independent to *mprF* alterations.^68^ Yet, other *mprF* mutations can confer reduced susceptibility to both VAN and DAP.^77,78^ Alterations in other genes such as *rpoB* could also yield a similar outcome.^69^ Importantly, there could also be implications of the *mprF*-mediated cross-resistance, such as cross-resistance with lipoglycopeptide dalbavancin.^79^

Exposure to sub-inhibitory cell-wall targeting antibiotic concentrations could lead to the development of a VISA phenotype,^87^ which includes autolysis tolerance. The VAN-3 isolate showed reduced autolytic activity similar to a VISA Mu50 strain known to have increased autolysis resistance.^70^ This is consistent with previous reports of mutations affecting the WalKR regulon resulting in a VISA phenotype,^7^ typically characterized by thickened cell wall and reduced autolysis.^70,88^ Suppression of the autolytic system could result from exposure to sub-inhibitory concentrations of cell wall inhibitors, likely to minimize damage to the cell wall.^89^ No significant difference was observed in the VAN-3 isolate DT, as noted for other *walK* mutants.^70^

This study has provided evidence that *in vitro* resistance selection emulated both the genomic and phenotypic changes acquired in the clinic. Although we have confirmed the role of certain genomic variants in contributing to resistance, further studies are needed to validate the role of novel mutations not previously reported, and the effects of multiple mutations in one isolate. Furthermore, this study has provided additional insight into cross-resistance between VAN and DAP mediated by certain mutations. Identifying variants at the genetic level can provide clues to the underlying mechanisms of these shifts in resistance profiles and provide information for alternative antibiotic treatment in the clinic. Additional experiments providing information on fitness, such as competitive growth assays, would be useful to generate more accurate predictions regarding the likelihood of that mutation being maintained within the population.^90^ This study has demonstrated the feasibility of using *in vitro* resistance selection to predict pathways impacted and in turn, potentially forecast the viability of new antibiotics entering the clinic.

## Supplementary Material

dlaf108_Supplementary_Data

## Supplementary data

Tables S1 to S5 is available as Supplementary data at *JAC-AMR* Online.
